# The Abuse of Tuberculin

**Published:** 1914-05-23

**Authors:** 


*To the Editor of* The Hospital.

### The Abuse of Tuberculin.

*To the Editor of* The Hospital.

Sir,—I notice that of late you have in your Journal devoted considerable space to the subject of tuberculosis. In particular, I was glad to see a useful summary of the Midhurst Report. It is surely time that a warning should be sounded against the indiscriminate use of tuberculin, especially in general practice.

The administration of this substance is becoming more and more limited even in the hands of experts, but is apparently being given by general practitioners in cases whose suitability is judged rather by financial than by clinical standards. The report of Dr. Bardswell gives sufficient evidence of the harmful qualities of tuberculin even when carefully administered, and it is unpleasant reading to learn that at least one member of our profession finds it consonant with his position to give a course of tuberculin through the post.

During the last two months I have come across three cases of what can scarcely be described otherwise than as tuberculin quackery. In one the patient had only basal signs, and received injections of tuberculin before the sputum had been examined or a record of the temperature obtained. The patient was able to pay good fees.

It is unnecessary to give details of other cases; but after reading the recent severe letters in your paper written by non-panellers on the panel "traitors," the thought arises in one's mind, Are all the virtues indeed concentrated amongst the non-panel free-lances? Some might more aptly be described as freebooters.—Yours faithfully,

May 18, 1914. A Tuberculosis Officer.

